# On bootstrap based variance estimation under fine stratification

**DOI:** 10.1371/journal.pone.0292256

**Published:** 2024-06-13

**Authors:** Alexis Habineza, Romanus Odhiambo Otieno, George Otieno Orwa, Nicholas Makumi

**Affiliations:** 1 Pan African University, Institute for Basic Sciences, Technology and Innovation (PAUSTI), Nairobi, Kenya; 2 Department of Statistics and Actuarial Sciences, Jomo Kenyatta University of Agriculture and Technology (JKUAT), Nairobi, Kenya; 3 Meru University of Science and Technology, Meru, Kenya; 4 Kibogora Polytechnic, Nyamasheke, Rwanda; CESi Engineering School: Ecole d’Ingenieurs CESi, FRANCE

## Abstract

The primary focus of all sample surveys is on providing point estimates for the parameters of primary interest, and also estimating the variance associated with those point estimates to quantify the uncertainty. Larger samples and important measurement tools can help to reduce the point estimates’ uncertainty. Numerous effective stratification criteria may be used in survey to reduce variance within stratum. In fine stratification design, the population is divided into numerous small strata, each containing a relatively small number of sampling units as one or two. This is done to ensure that certain characteristics or subgroups of the population are well-represented in the sample. But with many strata, the sample size within each stratum can become small, potentially resulting in higher errors and less stable estimates. The variance estimation process becomes difficult when we only have one unit per stratum. In that case, the collapsed stratum technique is the classical methods for estimating variance. This method, however, is biased and results in an overestimation of the variance. This paper proposes a bootstrap-based variance estimator for the total population under fine stratification, which overcomes the drawbacks of the previously explored estimation approach. Also, the estimator’s properties were investigated. A simulation study and practical application on survey of mental health organizations data were done to investigate properties of the proposed estimators. The results show that the proposed estimator performs well.

## 1 Introduction

Instead of enumerating the entire population, only the individuals in the sample survey are observed for the purpose of estimating population characteristics. The sample characteristics are used to approximate the population. The inaccuracy in such approximation is known as sampling error, and it is inherent and inescapable in all sampling designs. Nonetheless, when time and cost are considered, sampling results in considerable improvements. This is done to observe characteristics and subsequent handling of data.

A variety of sample selection designs are available, and careful selection will provide accurate and dependable estimates. Rough estimations of sample size *n* can be derived for each sampling strategy with the necessary degree of precision. The requirement for reliable estimates, generally for very small samples with limited survey resources, along with the various framing and sampling procedures, leads in a complex survey design that uses different sampling techniques. For units taken from a complex survey sampling design, the observed value of the variable of interest is neither independent nor identically distributed. In addition, survey processing strives to improve the quality and usability of survey data by eliminating estimation bias, meeting confidentiality rules and raising the survey’s complexity [1], the examples can be found in [2, 3].

The fundamental purpose of all sample surveys is to get a point estimate for the parameters of primary interest, and also estimating the variance associated with those point estimates to quantify the uncertainty. The significance of variance estimators and related standard errors stems from an estimator’s estimated variance being the most critical component of its quality.

Estimating the sampling variance can be extremely difficult because of the complex sample design, non-linear estimators, and survey processing effects [4]. Simple and exact analytic formulae for statistical variance estimations under various sample designs are offered in [5]. No closed-form formulae for estimating variances exist when sample designs are more complicated or deployed in multiple phases. Furthermore, sophisticated weighting mechanisms make the variance estimation formula of simple statistics like totals challenging, even with simple sample designs. When there is no accurate technique for unbiased computing estimates of point estimates’ standard errors, the only choice is to approximate the required quantities. A different approach is based on replication techniques to get results inside analytic techniques by applying simplified assumptions concerning the sample design or the statistic to be variance-estimated [6].

In a stratified sample design, the target population is divided into a finite number of subpopulations (strata) with homogeneous units that share at least one common trait, such as age, sex, educational or income level, geographical area and economic status among others. Homogeneous subpopulations are often defined by strata, thus reducing the total variance of the parameter of interest. Furthermore, because strata are supposed to be independent, stratification provides a flexible sampling technique per stratum, for example, simple random sampling (*SRS*) with or without replacement and systematic sampling. The samples in various strata are independent, each estimate and its related variance estimator are just the sums of the corresponding estimators inside each stratum. As a result, the difficulty of finding the proper variance estimator for a stratified single-stage sample is reduced to the problem of determining the optimal variance estimate for each stratum’s sampling designs [7–9]. Therefore, this study focuses on the specific issue of fine stratification design, in which the sample size per stratum is small as *n*_*i*_ = 1 or *n*_*i*_ = 2 primary sampling units (PSUs) selected using (*SRS*) without replacement.

The variance estimation process becomes difficult when we only have one unit per stratum. This scenario may arise if we have a highly fine stratification. Each stratum has a sample size greater than one, but only one responding unit exists; the sampling design itself imposes a single unit per stratum. For example, in [10] the new Canadian Health Measures Survey (CHMS) samples just a single PSU in one of its five strata, although CHMS estimates are required at the national level. Having a stratum with a single PSU is a fairly common problem. When there is only one PSU within a stratum, there is insufficient information with which to compute an estimate of that stratum’s variance. Some of the suggested solutions and their corresponding drawbacks are detailed and discussed in [9, 11–14].

The variance for two or three PSUs per stratum, on the other hand, is significant. A key technique for estimating the variance of an unknown parameter under fine stratification is to collapse neighboring strata to form pseudo-strata with such a higher number of PSUs and then estimate their variance.

For the first time, the method was introduced by [15], although it frequently overestimates the estimator’s variance. The collapsed stratum technique is the most commonly proposed strategy in the literature for dealing with this problem. The topic of collapsing strata for variance estimation with one unit per stratum is covered in [3, 5, 16, 17]. In either of these circumstances, it is challenging to calculate variances using one sampled unit per stratum directly. According to [15, 18], several auxiliary variables that are correlated well to the expected values of strata’s mean are recommended to minimize the bias of the variance estimator. Unfortunately, this type of useful auxiliary information may not be easily accessible for all strata. Mantel and his coauthor [10] presented to the CHMS a new technique on variance factors from distinct sample stages. Mosaferi [19] designed and constrained empirical Bayesian estimators for a one-unit variance per stratum sampling procedure. The author also compared one PSU per stratum design to two PSUs per stratum design, highlighting some of the inconsistencies of the proposed estimators due to the moment parameter estimation approach.

The collapsed stratum technique is the most commonly used strategy in the literature for dealing with this problem and its results in positive bias as discussed in section 2. By replacing a collapsed stratum estimator with a kernel-weighted stratum neighborhood and utilizing deviations from a fitted mean function, a nonparametric kernel-based technique for estimating variance was developed in [9]. They demonstrated the superiority of their method over the collapsed stratum variance estimator nonparametric using the United States Consumer Expenditure Survey. A major weakness with the use of nonparametric Kernel-based regression over a finite range, is the bias at boundary points. The bias of the estimators towards the boundary points decreases at the expense of increasing variance. The trade-off between Bias and Variance has thus remained a issue. Most of the proposed alternative methods for collapsed stratum variation are based on concomitant or auxiliary information; however, this type of desirable auxiliary information may not be readily available for all strata.

Fine stratification is a popular design as it allows the population to be divided into numerous small strata, each containing a relatively small number of sampling units. This is done to ensure that certain characteristics or subgroups of the population are well-represented in the sample and lead to more precise estimates for specific subgroups. Some examples include the Current Population Survey and National Crime Victimization Survey both conducted by the U.S. Census Bureau, and the National Survey of Family Growth conducted by the University of Michigan’s Institute for Social Research. Clearly, the fine stratification survey has proved useful in many applications as its point estimator is unbiased and efficient. In such situations, traditional variance estimation techniques may not perform well due to the limited number of observations in some strata.

This work suggests a bootstrap-based variance estimator for the total population under fine stratification as an additional method to overcome the inadequacies of previously explored estimate approaches. This method involves repeatedly resampling from the original sample with replacement to create multiple pseudo-samples. These pseudo-samples are used to compute the point estimator of interest (specifically the total) for each resample. The variance of the point estimator is then calculated based on the variability among these pseudo-estimates. The new method is detailed in section 4.

The paper structure is as follows: Section 2 offers a collapsing technique for variance estimation for the total population, section 3 presents non parametric kernel based variance estimation for the total population. Section 4 provides the bootstrap-based variance estimator development and the corresponding properties. Section 5 provides an empirical assessment of the findings, and Section 6 provides the conclusion.

## 2 Collapsing strata technique for variance estimation

When just one PSU is chosen per stratum or when only one PSU in a stratum participates, strata or PSUs are combined to generate pseudo or analytic strata for variance estimation. The number of PSUs in some sample designs can be extraordinarily enormous, especially in education and establishment surveys, where there can be thousands of first-stage units. In such cases, PSUs, strata, or both may be collapsed together.

Let the population total $t=\sum_{i=1}^{H}t_{i}$ be estimated by $\hat{t}=\sum_{i=1}^{H}{\hat{t}}_{i}=\sum_{i=1}^{H}\sum_{k\in U_{i}}\frac{y_{k}}{\pi_{k}}$, where ${\hat{t}}_{i}$ is unbiased estimator for the stratum total *t*_*i*_. Assuming a single element *k* is selected with inclusion probability *π*_*k*_ from the stratum, the *π*_*k*_ adds to unity in the stratum. In particular $\pi_{k}=\frac{1}{N_{i}}$ for all *k* if simple random selection is used. After pairing the strata, let *i* and *j* refer to the two strata in *i*^*th*^ and *j*^*th*^ pair such that *i* = 1, …, *H* and *j* = 1, …, *H*. We suppose that the value of the study variables *y*_*k*_ is observed without error for the unit *k* in the sample *s*. Our goal is to estimate the total population:

$$\begin{array}{c} t=\sum_{k\in U}y_{k}=\sum_{i=1}^{H}\;\sum_{k\in U_{i}}y_{k}=\sum_{i=1}^{H}\;t_{i} \end{array}$$ (1)

Let us define indicator variable *I*_*k*_ = 1 if *k* ∈ *s* and *I*_*k*_ = 0. If the second inclusion probability *π*_*kl*_ > 0 for all {k,l}∈U, the design is considered to be measurable, and the design variance admits an unbiased estimator as discussed in [20–22] and is given by:

$$\begin{array}{c} \hat{t}=\sum_{i=1}^{H}\;{\hat{t}}_{i}=\sum_{i=1}^{H}\;\sum_{k\in U_{i}}y_{k}\frac{I_{k}}{\pi_{k}} \end{array}$$ (2)

which is an unbiased estimation of *t*, and its variance is determined by

$$\begin{array}{c} \text{var}\left(\hat{t}\right)=\sum_{i=1}^{H}\;\text{var}({\hat{t}}_{i})=\sum_{i=1}^{H}\;V_{i} \end{array}$$ (3)

where *V*_*i*_ is defined by

$$\begin{array}{c} V_{i}=\sum_{k,l\in s_{i}}\sum (\pi_{kl}-\pi_{k}\pi_{l})\frac{y_{k}y_{l}}{\pi_{k}\pi_{l}} \end{array}$$ (4)

In [3, 9, 16] the collapsed stratum variance estimator is given by

$$\begin{array}{c} {\hat{V}}_{col}=\frac{1}{2}\sum_{i=1}^{H}{\left({\hat{t}}_{i}-\sum_{j=1}^{H}c_{j}\left(i\right){\hat{t}}_{j}\right)}^{2} \end{array}$$ (5)

where

$$\begin{array}{c} c_{j}\left(i\right)=\{\begin{array}{cc} 1 & \;\text{for}\;i,j:i\neq j\;\text{belong}\;\text{to}\;\text{the}\;\text{same}\;\text{collapsed}\;\text{stratum}\\ 0 & \text{otherwise} \end{array} \end{array}$$

The variance estimator given in (5) is design-biased, and its bias concerning the design is

$$\begin{array}{c} \text{Bias}[{\hat{V}}_{\text{col}}]=\frac{1}{2}\sum_{i=1}^{H}{\left(t_{i}-\sum_{j=1}^{H}c_{j}\left(i\right)t_{j}\right)}^{2} \end{array}$$ (6)

As shown in (6), the estimator in (5) has a positive bias, and the bias is small if the strata are successfully matched, in the sense that *t*_*i*_ ≈ *t*_*j*_ and *c*_*j*_(*i*) = 1. To retain the statistical properties, the pairing must be conducted irrespective of any sample knowledge. There is also a temporal, geographical, or other structure in population that uses fine stratification that may be employed in pairing just to minimize the difference *t*_*i*_ − *t*_*j*_ [6].

## 3 Nonparametric kernel based variance estimation

To reduce the bias in (5), the alternative method was introduced in [9], where, the binary function *c*_*j*_(*i*) in Eq (5) was replaced by the kernel weights defined in section (1.3) of [9]. The following equation, that is (7) provided the nonparametric variance estimator as an alternative to collapse variance estimator under fine stratification:

$$\begin{array}{c} {\hat{V}}_{ker}=\frac{1}{c_{d}}\sum_{i=1}^{H}{\left({\hat{t}}_{i}-\sum_{j=1}^{H}d_{j}\left(i\right){\hat{t}}_{j}\right)}^{2} \end{array}$$ (7)

The expectation variance of the estimator (7) is given by

$$\begin{array}{c} E[{\hat{V}}_{\text{ker}}]=c_{d}^{-1}\{\sum_{i=1}^{H}V_{i}(1-2d_{i}\left(i\right)+\sum_{j=1}^{H}d_{j}^{2}\left(i\right))+\sum_{i=1}^{H}{\left(\sum_{j=1}^{H}d_{j}\left(i\right)(t_{i}-t_{j})\right)}^{2}\} \end{array}$$ (8)

where the nonrandom normalizing constant, *c*_*d*_, depends on the kernel weights but not on the survey variables and it is defined by:

$$\begin{array}{c} c_{d}=\frac{1}{H}\sum_{i=1}^{H}(1-2d_{i}\left(i\right)+\sum_{j=1}^{H}d_{j}^{2}\left(i\right)) \end{array}$$ (9)

The estimator (7) has also a positive bias defined by

$$\begin{array}{c} Bias\left[{\hat{V}}_{ker}\right]=c_{d}^{-1}\sum_{i=1}^{H}{\left(\sum_{j=1}^{H}d_{j}\left(i\right)(t_{i}-t_{j})\right)}^{2} \end{array}$$ (10)

Therefore *c*_*d*_ was chosen to reduce the part of the bias due to *V*_*i*_’s if the *V*_*i*_’s are constant across strata.

## 4 Bootstrap-based variance estimator

The collapsed variance estimator defined in Eq (5) has non negative bias. Its alternative nonparametric kernel-based estimator defined by Eq (7), also suffer from the boundary bias. As it is known that most kernel smoother have boundary problems and require modifications at the boundary points. That is, towards the boundary points the bias of the estimators decreases at the cost of an increasing variance. It is assumed that for any collapsing, the contribution to the bias of the variance estimator from each pair of strata is known and non negative. Therefore, we are coming up with a methodology of developing the bootstrap-based variance estimator ${\hat{V}}_{boot}$ for a given set of *H* strata, that should be paired to reduce the bias of the variance estimator with no cost to the variance. When applying bootstrapping procedures this single unit can lead to a variety of issues.

The guiding principle in using the bootstrap method to stratified sampling designs is that the bootstrap replicate should itself be a stratified sample selected from the parent sample. However, in this paper, the parent sample usually has only one or two elements per stratum, which is meaningless in implementing resampling. Therefore, in this paper we combined a single unit at each stratum with the next smallest stratum to create the pseudo strata with at least two units, before applying the bootstrap process. Bootstrapping is applied after the process of collapsing the strata with the approximated characteristics which is the source of the bias. The bootstrap sampling is applied for the two groups of collapsed strata and for no collapsed strata by selecting a sample size of n* = 2 in each stratum. Therefore, the bootstrap bias corrector defined by Eq (13) is used to reduce the bias for the collapsed strata.

From (2), we define the bootstrap total population $t_{b}=\sum_{i=1}^{H}\sum_{k\in U_{i}}\;y_{k}^{*}\frac{1}{\pi_{k}}$ by using the replication variable $y_{k}^{*}$ in stratum population $U_{i}$. For a given *H*, over all *B* resamples across the stratum, the bootstrap estimator of total population *t*_*b*_ is calculated. We define the bias of an estimator ${\hat{t}}_{bj}$

$$\begin{array}{c} Bias\left({\hat{t}}_{bj}\right)=E\left({\hat{t}}_{bj}\right)-t_{bj}\approx O(\frac{1}{H}) \end{array}$$ (11)

A bootstrap-based approximation to this bias is given by

$$\begin{array}{c} {\hat{Bias}}_{B}\left({\hat{t}}_{bj}\right)=\frac{1}{B}\sum_{b=1}^{B}\left({\hat{t}}_{bj}-t_{bj}\right) \end{array}$$ (12)

where ${\hat{t}}_{bj}$ are copies of bootstrap of *t*_*bj*_.

This construction is also based on standard bootstrap thinking to replace the population with the sample’s empirical population. The following defines the bootstrap bias corrector:

$$\begin{array}{c} {\hat{a}}_{bj}={\hat{t}}_{bj}-{\hat{Bias}}_{B}\left({\hat{t}}_{bj}\right) \end{array}$$ (13)

Then from (7) we replace the weights *d*_*j*_(*i*) by the bootstrap bias corrector defined in (13), therefore, the bootstrap variance estimator under fine stratification is given by:

$$\begin{array}{c} {\hat{V}}_{boot}=\frac{1}{c_{b}}\sum_{i=1}^{H}{\left({\hat{t}}_{bi}-\sum_{j=1}^{H}{\hat{a}}_{bj}{\hat{t}}_{bj}\right)}^{2} \end{array}$$ (14)

where ${\hat{a}}_{bj}$ is the bootstrap bias corrector defined in (13) and *c*_*b*_ is the nonrandom normalizing constant depending on bootstrap bias corrector and is defined by:

### 4.1 Properties of the proposed estimator

The bootstrap variance estimator is judged based on design expectation, design variance, mean squared error, and a specific sampling design for the fixed finite population. Therefore, we are interested in finding the above estimators’ statistical properties about the sampling design. The design expectation of ${\hat{V}}_{boot}$ is given by:

$$\begin{array}{c} \begin{array}{cc} E[{\hat{V}}_{boot}] & =E[\frac{1}{c_{b}}\sum_{i=1}^{H}{\left({\hat{t}}_{bi}-\sum_{j=1}^{H}{\hat{a}}_{bj}{\hat{t}}_{bj}\right)}^{2}]\\ & =\frac{1}{c_{b}}\sum_{i=1}^{H}E{\left({\hat{t}}_{bi}-\sum_{j=1}^{H}{\hat{a}}_{bj}{\hat{t}}_{bj}\right)}^{2} \end{array} \end{array}$$ (15)

$$\begin{array}{c} E[{\hat{V}}_{boot}]=\frac{1}{c_{b}}\sum_{i=1}^{H}[V_{i}[1+\sum_{j=1}^{H}{\hat{a}}_{bj}^{2}-2\sum_{j=1}^{H}{\hat{a}}_{bj}]+{\left(t_{bi}-\sum_{j=1}^{H}a_{bj}t_{bj}\right)}^{2}] \end{array}$$ (16)

For more details consider the Appendix A in S1 File.

#### 4.1.1 The variance of the estimator

The design variance of ${\hat{V}}_{boot}$ is given by:

$$\begin{array}{c} Var\left({\hat{V}}_{boot}\right)=E[{\hat{V}}_{boot}^{2}]-{\left(E[{\hat{V}}_{boot}]\right)}^{2} \end{array}$$

Hence the Variance of ${\hat{V}}_{boot}$ is given by:

$$\begin{array}{c} Var\left({\hat{V}}_{boot}\right)=\frac{1}{c_{b}^{2}}\sum_{i=1}^{H}V_{i}^{2}[1-4\sum_{j=1}^{H}{\hat{a}}_{bj}^{2}+\sum_{j=1}^{H}{\hat{a}}_{bj}^{4}] \end{array}$$ (17)

The prove is on Appendix B in S1 File.

#### 4.1.2 Mean squared error of the estimator

The design mean squared error of our estimator is expressed in terms of bias and variance. However, for the sake of simplicity, it is easily seen that $V_{boot}=Var\left(\hat{t}\right)$ and the bias of ${\hat{V}}_{boot}$ is

$$\begin{array}{c} E[{\hat{V}}_{boot}]-V_{boot}=E[{\hat{V}}_{boot}]-Var\left(\hat{t}\right)=\frac{1}{c_{b}}\sum_{i=1}^{H}V_{i}[1+\sum_{j=1}^{H}{\hat{a}}_{bj}^{2}-2\sum_{j=1}^{H}{\hat{a}}_{bj}] \end{array}$$

Accordingly, the design mean squared error of our estimator is given as:

$$\begin{array}{c} \begin{array}{cc} MSE({\hat{V}}_{boot}) & =Var\left({\hat{V}}_{boot}\right)+{\left(E[{\hat{V}}_{boot}]-V_{boot}\right)}^{2}=Var\left({\hat{V}}_{boot}\right)+{\left(E[{\hat{V}}_{boot}]-Var\left(\hat{t}\right)\right)}^{2}\\ & =\frac{1}{c_{b}^{2}}\sum_{i=1}^{H}V_{i}^{2}[1-4\sum_{i=1}^{H}{\hat{a}}_{bj}^{2}+\sum_{i=1}^{H}{\hat{a}}_{bj}^{4}]\\ & +{\left(\frac{1}{c_{b}}\sum_{i=1}^{H}V_{i}[1+\sum_{j=1}^{H}{\hat{a}}_{bj}^{2}-2\sum_{j=1}^{H}{\hat{a}}_{bj}]\right)}^{2}\\ & =\frac{1}{c_{b}^{2}}\sum_{i=1}^{H}V_{i}^{2}[2+\sum_{j=1}^{H}({\hat{a}}_{bj}^{4}-3{\hat{a}}_{bj}^{2}-2{\hat{a}}_{bj})] \end{array} \end{array}$$ (18)

The *MSE* of the estimators could be simply used for the efficiency comparison, which includes the information of estimator variance and bias. By comparing Eqs (17) and (18), it is easy to see that both relations are approximately related thus the bias of our estimator is expected to be small.

## 5 Simulation study

### 5.1 Unconditional simulation study

In the simulation, we investigate the behavior of bootstrap-based variance estimators as compared to collapsed variance estimator and nonparametric kernel-based variance estimator. These are estimated under fine stratification at different strata, bandwidth, and standard deviation error values. For the nonparametric kernel based variance estimator in (7), the Epanechnikov kernel function,

$$\begin{array}{c} K\left(s\right)=\frac{3}{4}(1-s^{2})I_{\left\{|s|\leq 1\right\}} \end{array}$$

is used and bandwidths are chosen as 1/*H* < *h* < 2/*H* to yield smallest possible nonempty kernel window therefore *h* = {0.025, 0.015, 0.045, 0.0055, 0.0075} has been considered, the detailed discussion seen in [9]. The population *x*_*k*_ is generated as a set of uniform (0, 1) random variables that are distributed independently and identically. A stratified finite population was created with eight survey variables of interest with *H* evenly sized strata of size *N*_*i*_ = *N*/*H* and *x*_*i*_ = *i*/*H* for stratum *i*. During this simulation, 1000 bootstrap samples were used to assess the estimator’s quality. Then, the simulated data was stratified so that each stratum can have two primary sample units, and then evaluated the variance using the three variance estimators specified by Eqs (5), (7) and (14). Three possible values for standard deviation were considered: *σ* = 0, *σ* = 0.25, and *σ* = 0.5. For each of the seven variables, the population is *N* = 3000. Simple random sampling without replacement is used to create samples with stratum sizes of *H* = 50, *H* = 100, and *H* = 200 because fine stratification allows deep stratification, therefore a larger number of strata have been created and, in all cases, we consider *H* = 30 to be collapsed. Increasing sample size has the same effect as lowering standard deviation. Therefore, estimators’ design-averaged performance can be evaluated as the population is kept constant during these 1000 bootstrap samples. The design bias, variance, and mean squared error were calculated, and the mean functions were assumed for the eight variables of interest to be

$$\begin{array}{c} \mu_{*}^{\left(\ell \right)}\left(x\right)=2\frac{\mu_{\ell }\left(x\right)-{\text{min}}_{x\in [0,1]}\mu_{\ell }\left(x\right)}{{\text{max}}_{x\in [0,1]}\mu_{\ell }\left(x\right)-{\text{min}}_{x\in [0,1]}\mu_{\ell }\left(x\right)} \end{array}$$ (19)

This indicates that for each of the first seven mean functions, the lowest is zero and the maximum is two. In all cases, the population values $y_{k}^{\ell },\left(\ell =1,...,7\right)$ are generated from the mean functions by adding i.i.d *N*(0, *σ*^2^) errors. That is;

$$\begin{array}{c} y_{k}^{\left(\ell \right)}=\mu_{*}^{\left(\ell \right)}(x_{i})+\sigma e_{k}\;\text{for}\;k\in U_{i} \end{array}$$ (20)

so that, the total is given by

$$\begin{array}{c} t_{i}^{\left(\ell \right)}=\frac{N}{H}\mu_{*}^{\left(\ell \right)}(x_{i})+\sigma \sum_{k\in U_{i}}e_{k}=\mu^{\left(\ell \right)}(x_{i})+\epsilon_{i}. \end{array}$$ (21)

where the mean functions are defined as:

$$\begin{array}{c} \begin{array}{cc} \;\text{Linear:}\; & \mu_{1}\left(x\right)=1+2\left(x-0.5\right)\\ \;\text{Quadratic:}\; & \mu_{2}\left(x\right)=1+2{\left(x-0.5\right)}^{2}\\ \;\text{Bump:}\; & \mu_{3}\left(x\right)=1+2\left(x-0.5\right)+\text{exp}(-200{\left(x-0.5\right)}^{2})\\ \;\text{Jump:}\; & \mu_{4}\left(x\right)=\{1+2\left(x-0.5\right)I_{\left\{x\leq 0.65\right\}}\}+0.65I_{\left\{x>0.65\right\}},\\ \;\text{Exponential:}\; & \mu_{6}\left(x\right)=\text{exp}\left(-8x\right)\\ \;\text{Cycle1:}\; & \mu_{7}\left(x\right)=2+\text{sin}\left(2\pi x\right)\\ \;\text{Cycle4:}\; & \mu_{8}\left(x\right)=2+\text{sin}\left(8\pi x\right) \end{array} \end{array}$$ (22)

with *x* ∈ [0, 1]. The above means reflect a range of correct and incorrect model specifications for the estimators under evaluation [22]. As the anticipated model is accurately defined, *μ*_1_, is the preferable estimator. As a result, it’s fascinating to analyze how much efficiency is lost by assuming a smooth rather than underlying linear model. The remaining mean functions deviate from the linear model in various ways. The trend for *μ*_2_ is quadratic and assumed linear model would be incorrect for the whole range of *x*_*k*_, but appropriate locally. Except for a bump presenting a small portion of the *x*_*k*_, range, the function *μ*_3_ is linear across most of its range. The smoothness of the mean function *μ*_4_ is not present. The function *μ*_6_ is a sinusoid that completes one full cycle on [0, 1], whereas the function *μ*_7_ completes four full cycles and *μ*_5_ is exponential as discussed in [8, 9, 23].

When *V*_*i*_ = 0, meaning that $var(\hat{t})=0$, Table 1 illustrates the precise biases of the variance estimators. The conclusions described here apply to any design because the *t*_*i*_ values, the kernel, and *H* are the primary determinants of the variance estimators’ expectation and bias in this case. Compared to the collapsed stratum variance estimator and the non-parametric kernel based variance estimator, the suggested bootstrap-based variance estimator has a substantially less bias for each response variable.

**Table 1:** The exact bias of ${\hat{V}}_{col}$, ${\hat{V}}_{ker}$ and ${\hat{V}}_{boot}$ for *σ* = 0.

scenarios	estimator	line	quad	bump	jump	expo	cycle1	cycle4
*H* = 50	${\hat{V}}_{col}$	99.10	78.57	116.43	93.224	28.22	108.71	117.08
*h* = 0.025	${\hat{V}}_{ker}$	3.09	315.97	142.59	3800.16	128.04	49.96	1282.3
	${\hat{V}}_{boot}$	0.037	0.048	0.034	0.027	0.076	0.057	0.029
*H* = 100	${\hat{V}}_{col}$	12.18	8.42	14.78	11.29	2.83	13.52	13.80
*h* = 0.015	${\hat{V}}_{ker}$	1.14	1.74	8.21	5.54	7.33	1.52	2.97
	${\hat{V}}_{boot}$	0.019	0.033	0.017	0.016	0.023	0.019	0.030
*H* = 200	${\hat{V}}_{col}$	1.51	0.97	1.86	1.39	0.32	1.69	1.70
*h* = 0.0055	${\hat{V}}_{ker}$	0.086	0.87	0.46	791.05	0.41	3.56	31.94
	${\hat{V}}_{boot}$	0.018	0.014	0.011	0.017	0.0086	0.024	0.025

Table 2 compares the bias of the three estimators for standard deviation error values other than zero.

**Table 2:** The bias comparison of ${\hat{V}}_{col}$, ${\hat{V}}_{ker}$ and ${\hat{V}}_{boot}$ estimators.

Cases	estimator	line	quad	bump	jump	expo	cycle1	cycle4
*H* = 50	${\hat{V}}_{col}$	99.10	78.57	116.43	93.224	28.22	108.71	117.08
*h* = 0.025	${\hat{V}}_{ker}$	3.09	315.97	142.59	3800.16	128.04	49.96	1282.3
*σ* = 0.25	${\hat{V}}_{boot}$	0.037	0.048	0.034	0.027	0.076	0.057	0.029
*H* = 100	${\hat{V}}_{col}$	12.18	8.42	14.78	11.29	2.83	13.52	13.80
*h* = 0.015	${\hat{V}}_{ker}$	1.14	1.74	8.21	5.54	7.33	1.52	2.97
*σ* = 0.25	${\hat{V}}_{boot}$	0.019	0.033	0.017	0.016	0.023	0.019	0.030
*H* = 200	${\hat{V}}_{col}$	3.23	1.94	3.71	2.77	0.63	3.37	2.30
*h* = 0.0055	${\hat{V}}_{ker}$	0.028	0.97	0.46	791.05	0.41	0.15	0.03
*σ* = 0.25	${\hat{V}}_{boot}$	0.017	0.001	0.021	0.011	0.002	0.021	0.020
*H* = 50	${\hat{V}}_{col}$	19.21	15.71	23.28	18.86	5.64	21.74	23.42
*h* = 0.025	${\hat{V}}_{ker}$	16.93	15.90	71.56	19.01	64.18	25.26	56.48
*σ* = 0.5	${\hat{V}}_{boot}$	0.02	0.04	0.033	0.06	0.61	0.04	0.08
*H* = 100	${\hat{V}}_{col}$	2.44	1.68	2.95	2.25	0.56	2.70	2.76
*h* = 0.015	${\hat{V}}_{ker}$	3.73	1.28	6.3	13.48	5.44	2.10	4.33
*σ* = 0.5	${\hat{V}}_{boot}$	0.03	0.04	0.02	0.03	0.08	0.03	0.03
*H* = 200	${\hat{V}}_{col}$	0.30	0.19	0.37	0.18	0.06	0.33	0.34
*h* = 0.0055	${\hat{V}}_{ker}$	0.18	0.41	0.24	8.02	0.87	0.21	0.39
*σ* = 0.5	${\hat{V}}_{boot}$	0.03	0.05	0.04	0.03	0.06	0.03	0.02

The Table 3 compare both the *RMSE* of the non parametric kernel based variance estimator ${\hat{V}}_{ker}$ and the bootstrap-based variance estimator ${\hat{V}}_{boot}$.

**Table 3:** The performance of the estimators based on RMSE.

*H*	*h*	*σ*	*RMSE*	Line	Quad	Bump	Jump	Expo	Cycle1	Cycle4
50	0.025	0.25	${\hat{V}}_{col}$	201.39	161.26	136.12	188.67	63.17	221.62	238.07
			${\hat{V}}_{ker}$	162.01	158.07	71.74	190.41	61.03	210.04	154.41
			${\hat{V}}_{boot}$	3.13	3.45	3.86	2.50	3.84	3.33	2.80
100	0.015	0.25	${\hat{V}}_{col}$	30.05	24.64	35.33	26.71		34.43	34.91
			${\hat{V}}_{ker}$	22.32	11.78	18.64	19.01	7.91	32.10	16,27
			${\hat{V}}_{boot}$	0.76	1.40	0.86	0.64	2.28	1.03	1.02
200	0.0055	0.25	${\hat{V}}_{col}$	9.23	9.16	10.27	7.57	6.68	11.13	11.09
			${\hat{V}}_{ker}$	2.40	2.43	2.49	13.34	2.32	1.98	7.34
			${\hat{V}}_{boot}$	0.95	0.69	0.43	0.32	0.56	0.65	0.52
50	0.025	0.5	${\hat{V}}_{col}$	199.39	161.27	236.13	188.67	63.17	221.63	231.08
			${\hat{V}}_{ker}$	175.50	158.07	171.58	149.05	60.16	202.66	196.46
			${\hat{V}}_{boot}$	11.84	37.01	28.66	11.51	12.78	13.69	8.06
100	0.015	0.5	${\hat{V}}_{col}$	30.05	24.64	35.33	26.71	14.72	24.43	24.90
			${\hat{V}}_{ker}$	22.59	19.71	27.83	19.02	8.01	6.08	16.74
			${\hat{V}}_{boot}$	17.61	2.81	1.37	2.51	3.69	1.56	2.13
200	0.0055	0.5	${\hat{V}}_{col}$	9.24	9.18	10.29	7.58	6.68	11.16	11.18
			${\hat{V}}_{ker}$	5.07	3.02	2.47	13.35	5.99	2.66	2.58
			${\hat{V}}_{boot}$	1.81	1.24	1.03	0.81	1.87	1.17	1.34
100	0.045	0.25	${\hat{V}}_{col}$	30.05	24.64	35.33	26.71	14.72	34.43	34.90
			${\hat{V}}_{ker}$	19.77	15.30	23.85	17.57	11.09	8.64	19.30
			${\hat{V}}_{boot}$	1.15	1.91	0.85	1.07	0.43	4.18	1.26
200	0.0075	0.25	${\hat{V}}_{col}$	9.23	9.17	10.28	7.57	6.67	11.15	11.18
			${\hat{V}}_{ker}$	11.05	5.24	4.25	7.19	3.23	10.34	7.26
			${\hat{V}}_{boot}$	0.17	0.47	0.51	0.86	0.36	0.86	0.74
100	0.045	0.5	${\hat{V}}_{col}$	30.05	24.64	34.35	26.71	14.72	34.43	34.91
			${\hat{V}}_{ker}$	19.95	10.12	10.97	17.57	11.12	8.75	19.13
			${\hat{V}}_{boot}$	1.67	0.73	1.27	1.14	3.26	1.28	2.16
200	0.0075	0.5	${\hat{V}}_{col}$	9.21	9.11	10.31	6.9	7.01	10.91	11.06
			${\hat{V}}_{ker}$	2.56	1.08	2.62	7.59	2.31	2.66	7.34
			${\hat{V}}_{boot}$	0.96	1.13	0.92	0.64	1.28	0.70	1.01

The suggested bootstrap variance estimator has a smaller *RMSE* than the collapsed stratum variance estimator, frequently significantly lower in every scenario studied. At each value of *H*, ${\hat{V}}_{boot}$ outperforms ${\hat{V}}_{col}$ because it has a more negligible bias; at higher strata, the variability of the two estimators is essentially comparable.

The Table 4 compares both the *Bias* of the non parametric kernel based variance estimator ${\hat{V}}_{ker}$ and the bootstrap-based variance estimator ${\hat{V}}_{boot}$. The simulation results in illustrate the difference in bias between the two variance estimators. The findings reinforce the preference of ${\hat{V}}_{boot}$ especially for higher number of strata *H*.

**Table 4:** The performance of the estimators based on biases.

*H*	*h*	*σ*	*Bias*	Line	Quad	Bump	Jump	Expo	Cycle1	Cycle4
50	0.025	0.25	${\hat{V}}_{col}$	189.20	157.14	223.85	186.44	56.44	217.42	234.16
			${\hat{V}}_{ker}$	155.53	108.06	73.84	92.84	46.03	125.45	156.35
			${\hat{V}}_{boot}$	15.52	36.66	19.58	17.95	8.64	11.66	19.50
100	0.015	0.25	${\hat{V}}_{col}$	24.40	16.18	29.56	22.59	5.67	27.03	26.70
			${\hat{V}}_{ker}$	1.25	11.51	13.59	18.90	4.58	11.32	14.28
			${\hat{V}}_{boot}$	1.05	1.91	1.64	0.25	0.46	0.02	0.03
200	0.0055	0.25	${\hat{V}}_{col}$	3.02	4.09	3.70	2.78	0.63	3.31	3.26
			${\hat{V}}_{ker}$	1.84	1.95	2.46	7.99	2.29	2.11	2.79
			${\hat{V}}_{boot}$	0.04	0.05	0.03	0.02	0.05	0.02	0.03
50	0.025	0.5	${\hat{V}}_{col}$	98.21	57.15	138.86	168.45	56.44	172.70	163.71
			${\hat{V}}_{ker}$	69.54	49.07	132.43	139.09	125.07	46.16	44.64
			${\hat{V}}_{boot}$	11.18	37.01	86.64	115.13	18.74	12.09	18.06
100	0.015	0.5	${\hat{V}}_{col}$	24.37	16.84	29.56	22.59	8.57	27.04	27.60
			${\hat{V}}_{ker}$	3.72	2.11	12.21	19.02	7.99	21.03	16.41
			${\hat{V}}_{boot}$	1.44	1.08	1.34	2.29	1.12	1.61	1.24
200	0.0055	0.5	${\hat{V}}_{col}$	3.02	4.12	3.71	2.77	1.63	3.37	3.39
			${\hat{V}}_{ker}$	2.46	1.94	2.47	7.97	2.31	2.12	2.85
			${\hat{V}}_{boot}$	0.044	0.048	0.051	0.0.029	0.015	0.032	0.031
100	0.045	0.25	${\hat{V}}_{col}$	24.37	16.84	29.56	22.59	5.67	27.04	27.60
			${\hat{V}}_{ker}$	2.58	9.89	9.67	16.51	4.17	8.64	4.46
			${\hat{V}}_{boot}$	1.42	1.83	2.65	0.92	1.48	1.69	3.70
200	0.0075	0.25	${\hat{V}}_{col}$	3.02	1.94	3.71	2.77	0.63	3.37	3.39
			${\hat{V}}_{ker}$	0.03	0.97	0.49	7.19	0.42	0.15	3.27
			${\hat{V}}_{boot}$	0.007	0.02	0.05	0.08	0.07	0.03	0.07
100	0.045	0.5	${\hat{V}}_{col}$	224.37	16.84	29.57	22.59	5.67	27.04	27.60
			${\hat{V}}_{ker}$	3.71	9.23	10.78	15.46	3.11	7.97	14.31
			${\hat{V}}_{boot}$	2.64	2.24	4.18	1.43	1.80	4.27	2.36
200	0.0075	0.5	${\hat{V}}_{col}$	3.18	4.09	3.87	2.23	1.71	3.90	3.51
			${\hat{V}}_{ker}$	1.84	4.11	2.46	7.43	2.29	2.11	2.63
			${\hat{V}}_{boot}$	0.027	0.016	0.010	0.023	0.081	0.050	0.044

### 5.2 Conditional simulation study

**Fig 1:**
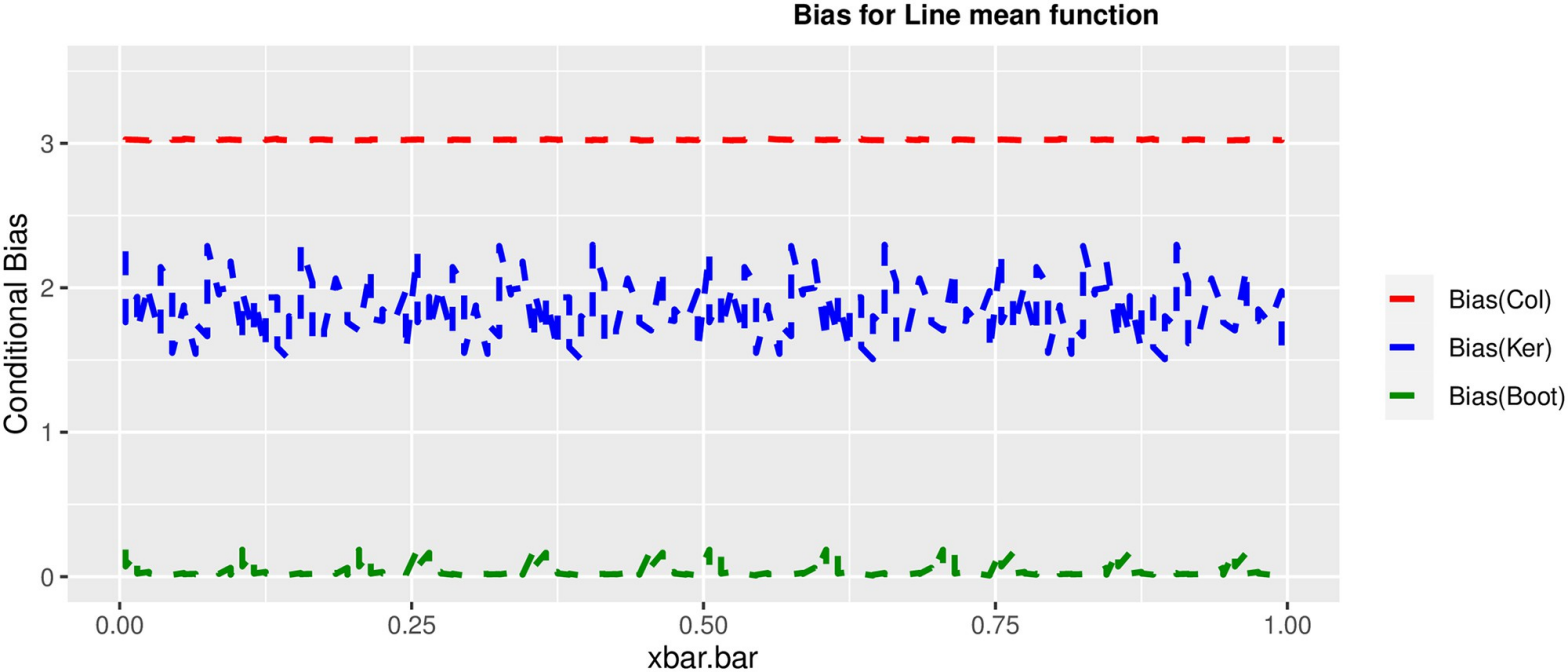
Comparison based on the biases of the 3 estimators for linear mean function.

**Fig 2:**
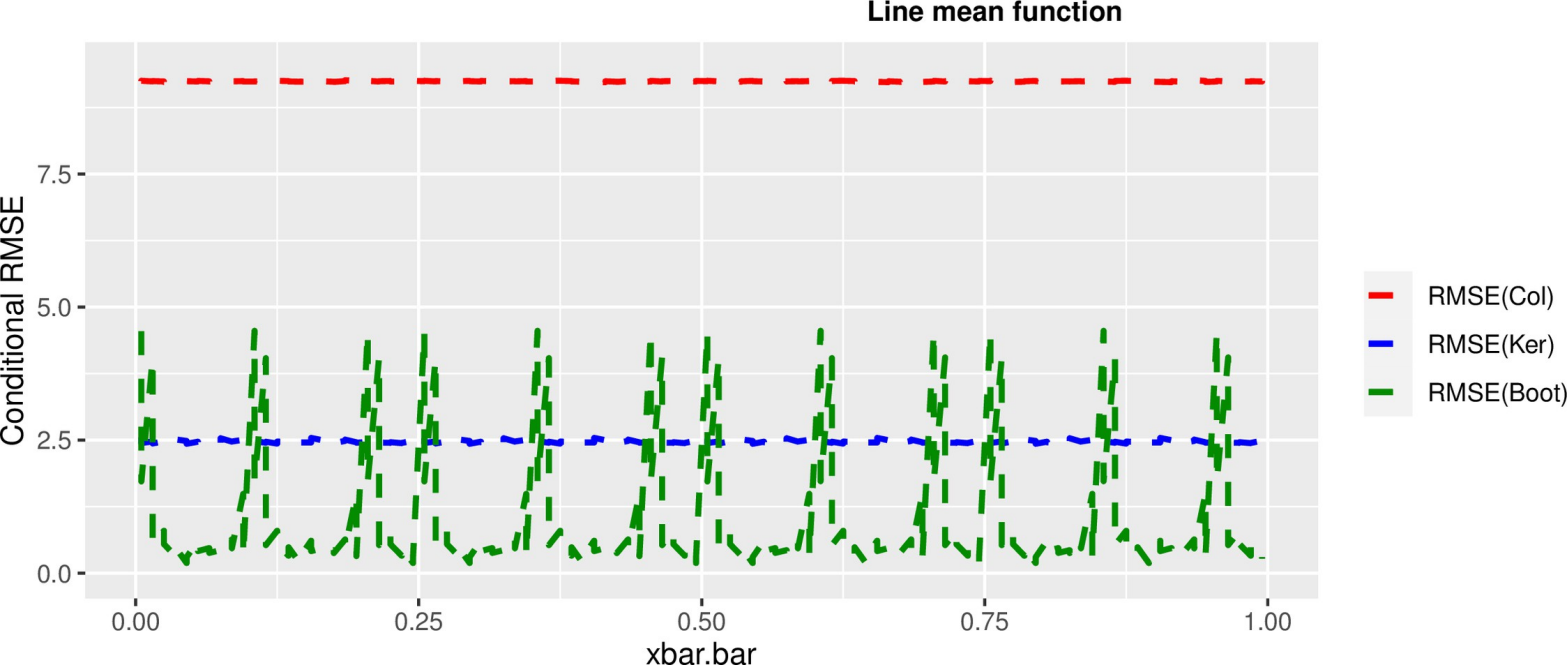
Comparison based on RMSE of the 3 estimators for linear mean function.

**Fig 3:**
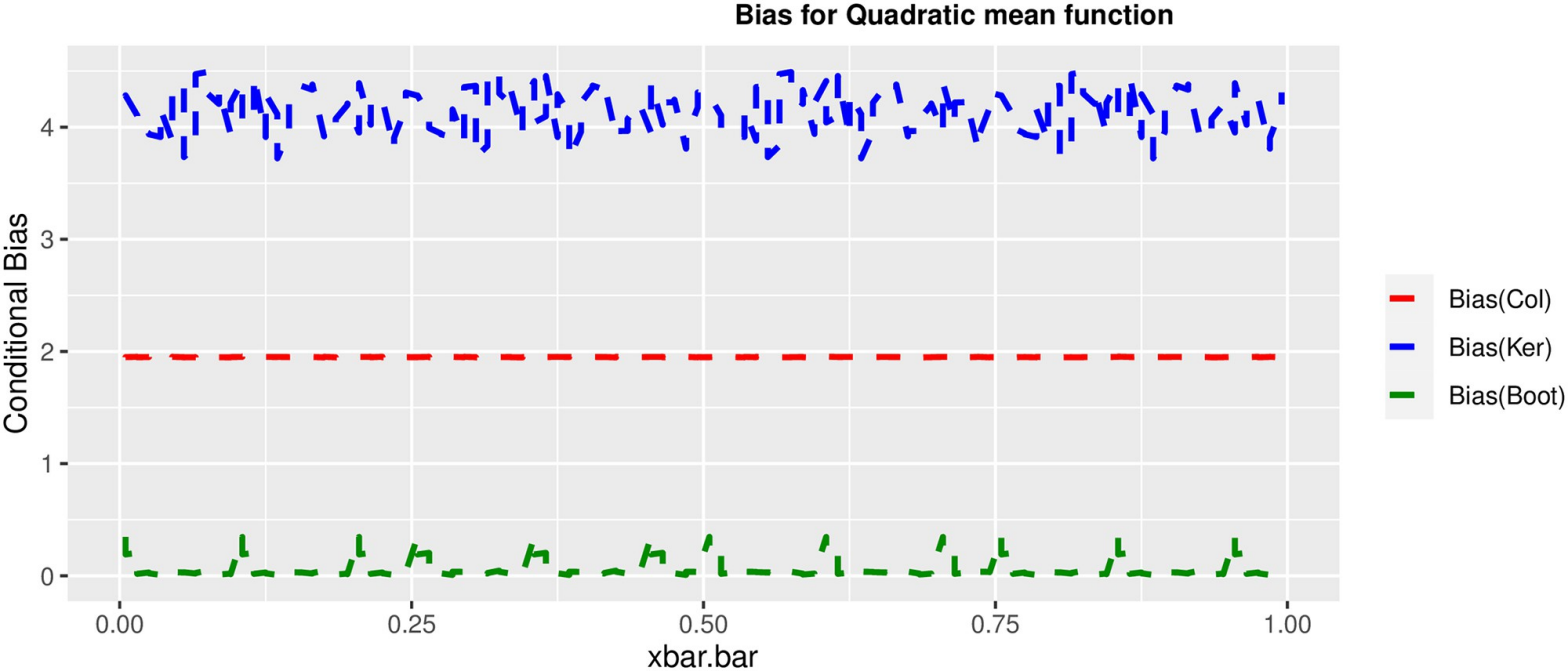
Comparison based on biases of the 3 estimators for quadratic mean function.

**Fig 4:**
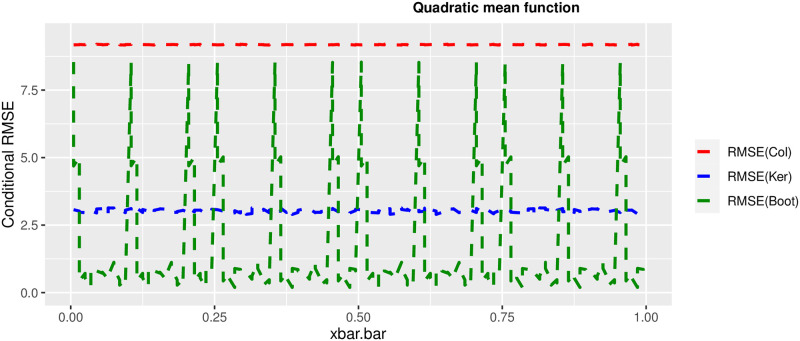
Comparison based on RMSE of the 3 estimators for quadratic mean function.

**Fig 5:**
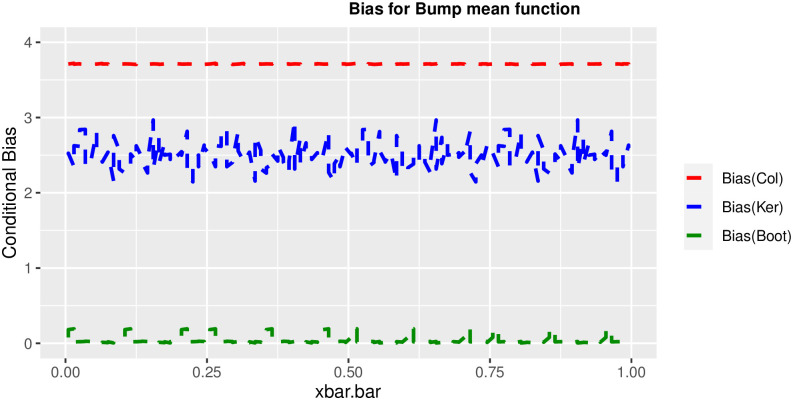
Comparison based on biases of the 3 estimators for bump mean function.

**Fig 6:**
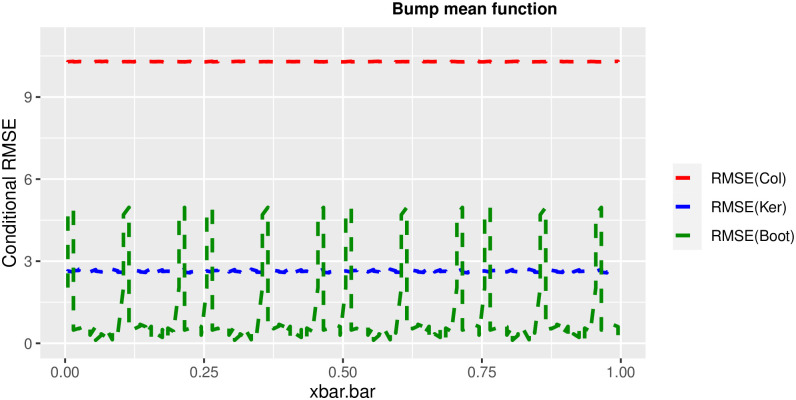
Comparison based on RMSE of the 3 estimators for bump mean function.

**Fig 7:**
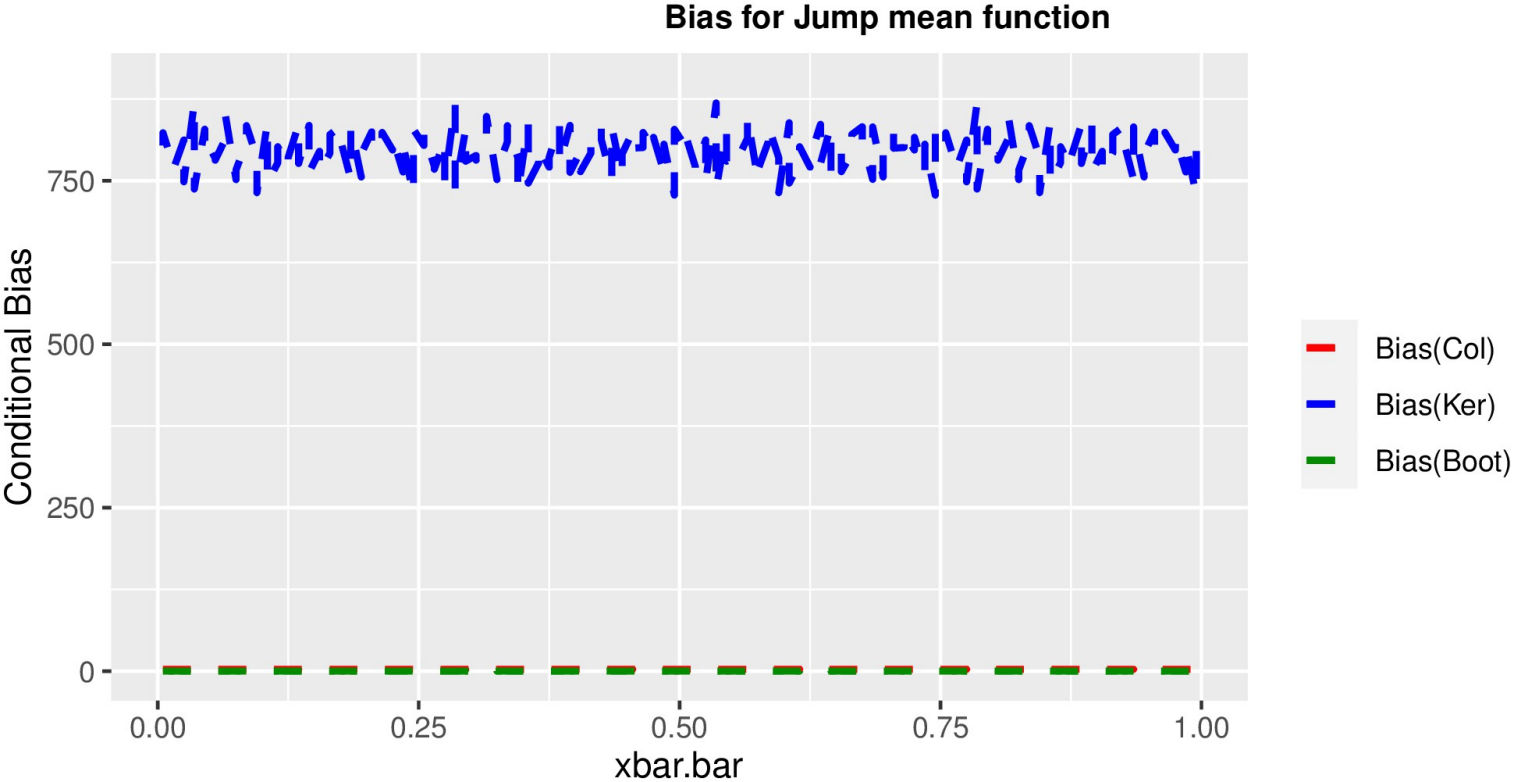
Comparison based on biases of the 3 estimators for jump mean function.

**Fig 8:**
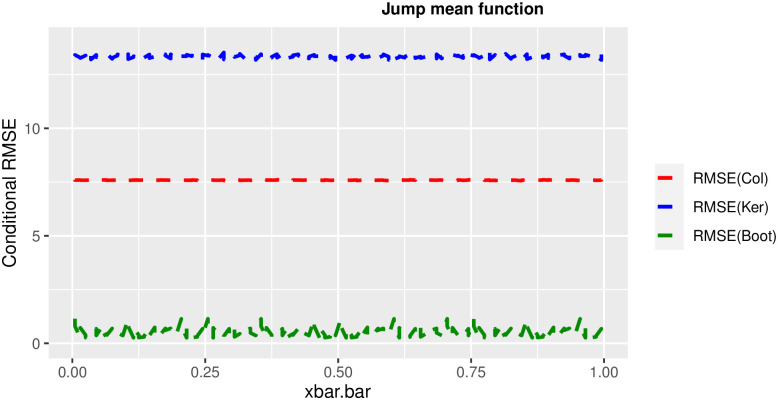
Comparison based on RMSE of the 3 estimators for jump mean function.

**Fig 9:**
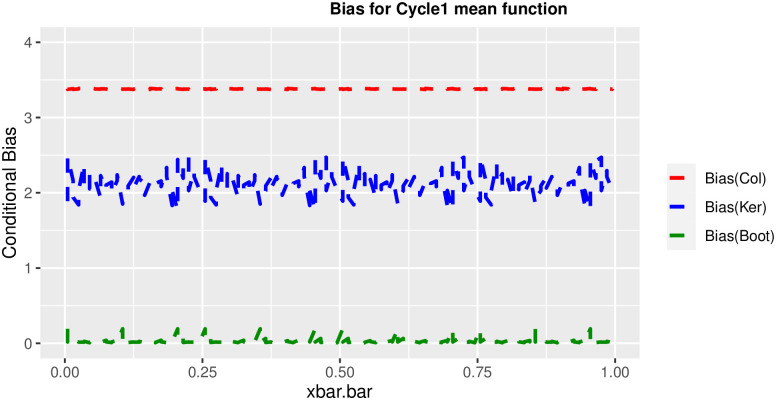
Comparison based on biases of the 3 estimators for cycle1 mean function.

**Fig 10:**
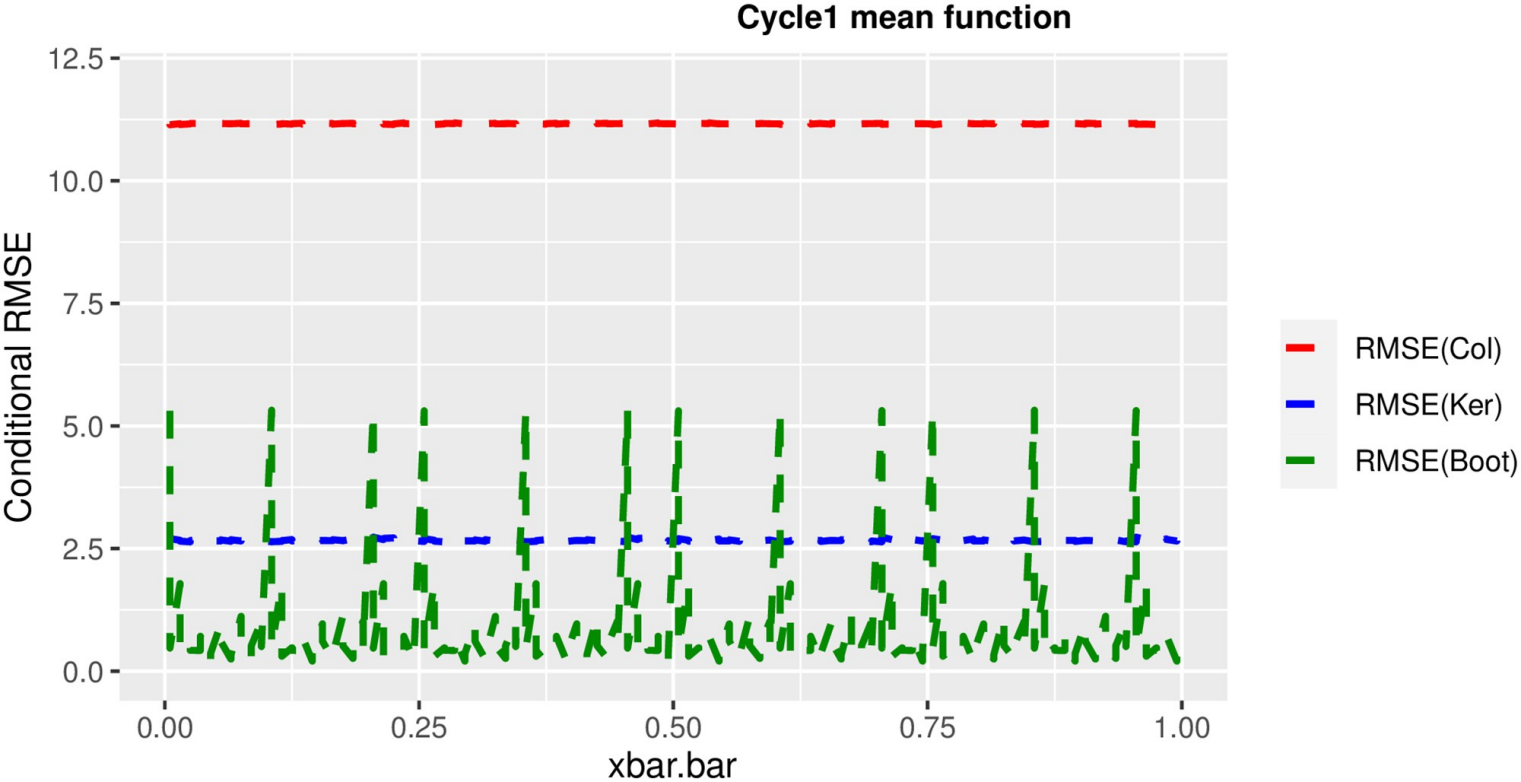
Comparison based on RMSE of the 3 estimators for cycle1 mean function.

**Fig 11:**
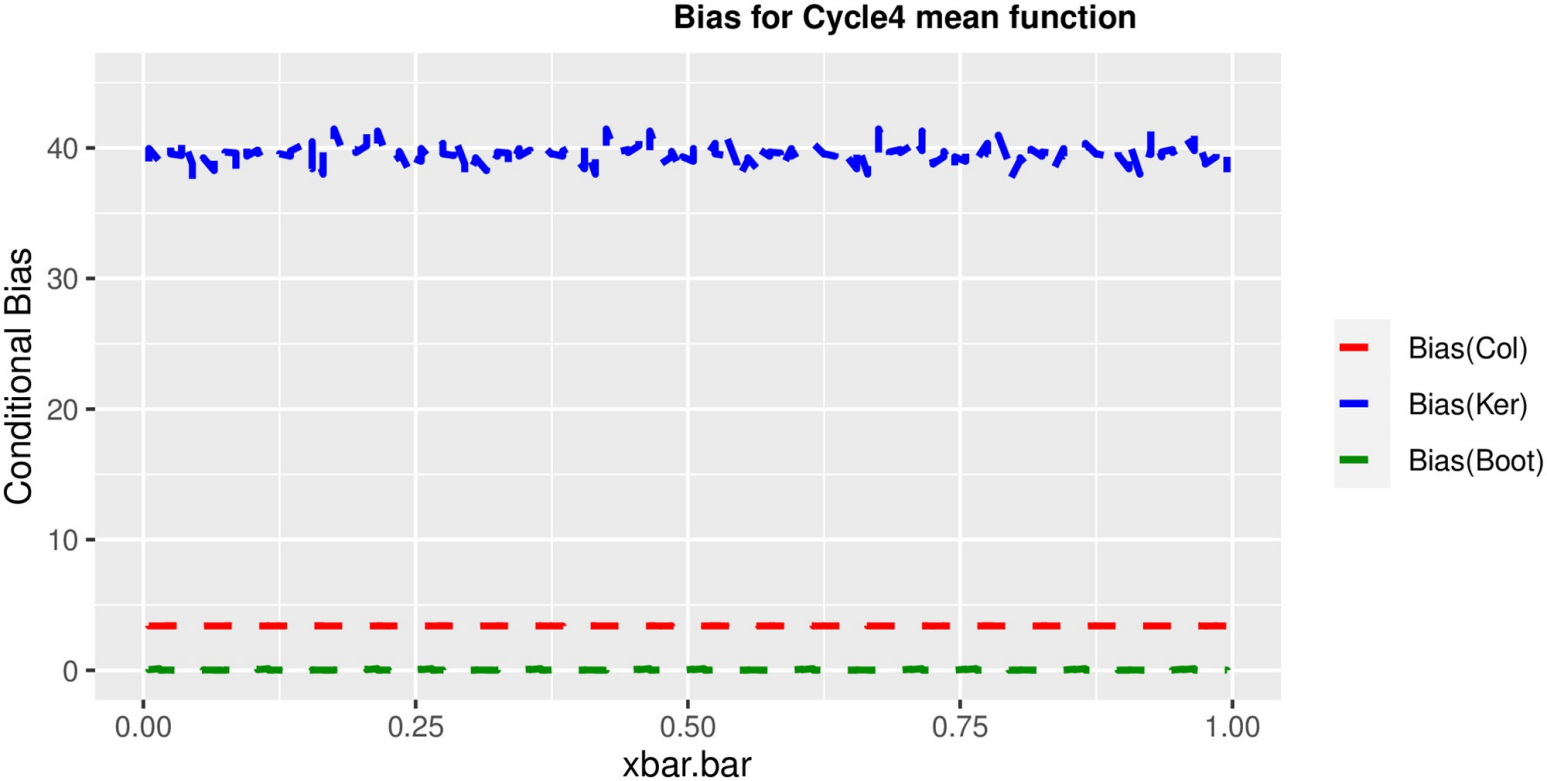
Comparison based on biases of the 3 estimators for cycle4 mean function.

**Fig 12:**
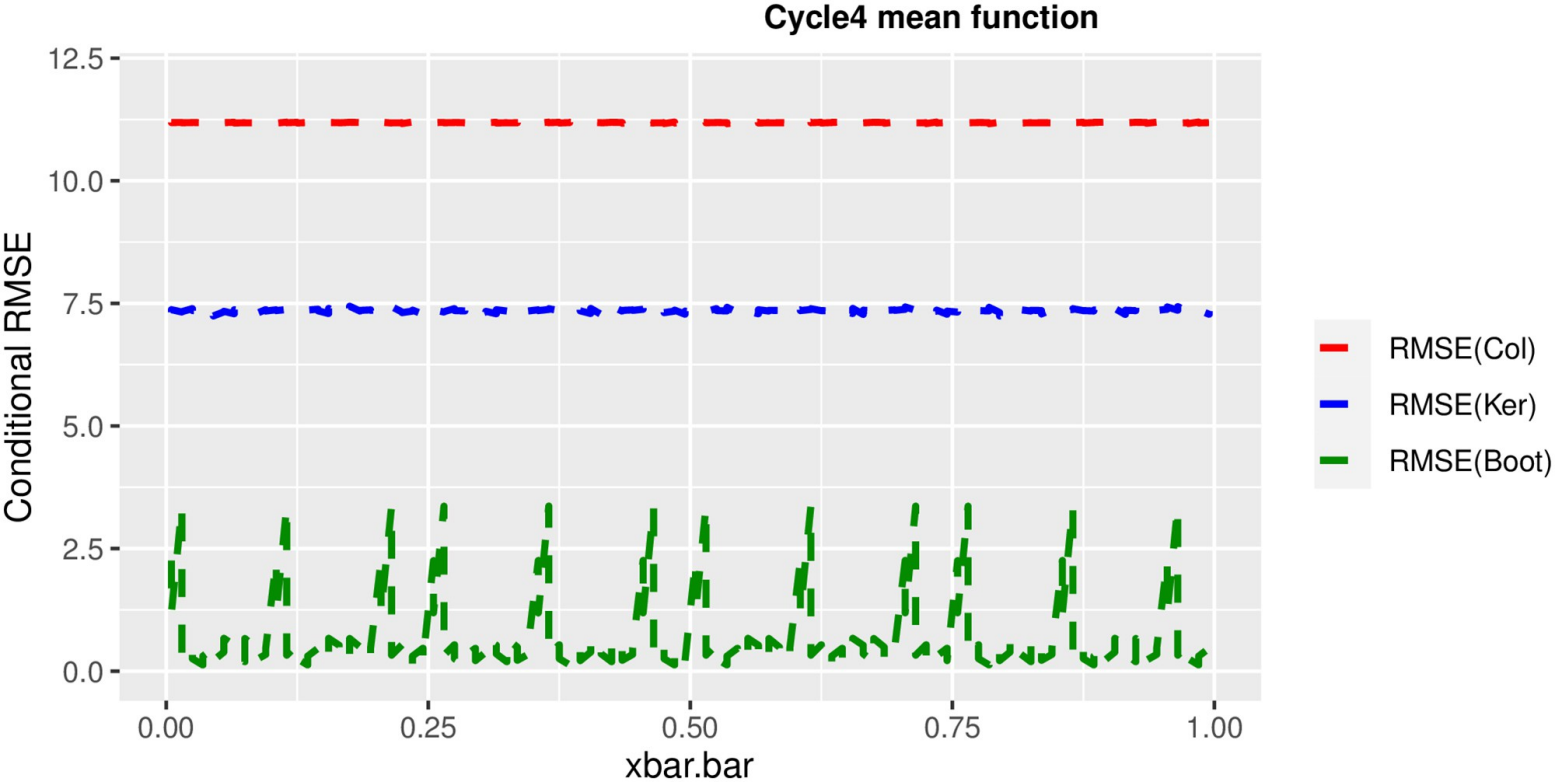
Comparison based on RMSE of the 3 estimators for cycle4 mean function.

**Fig 13:**
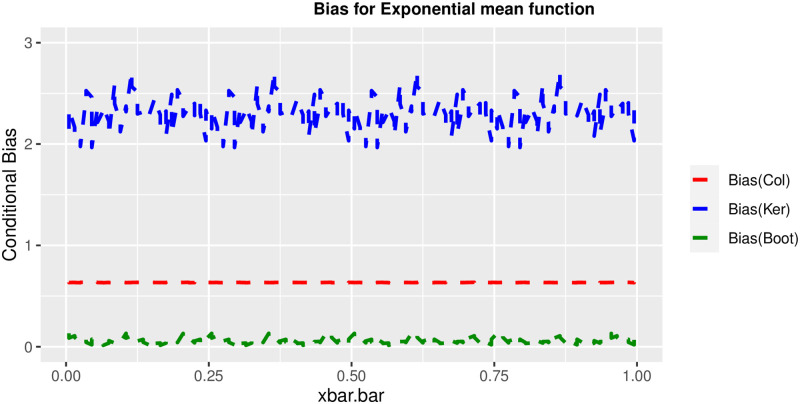
Comparison based on biases of the 3 estimators for exponential mean function.

**Fig 14:**
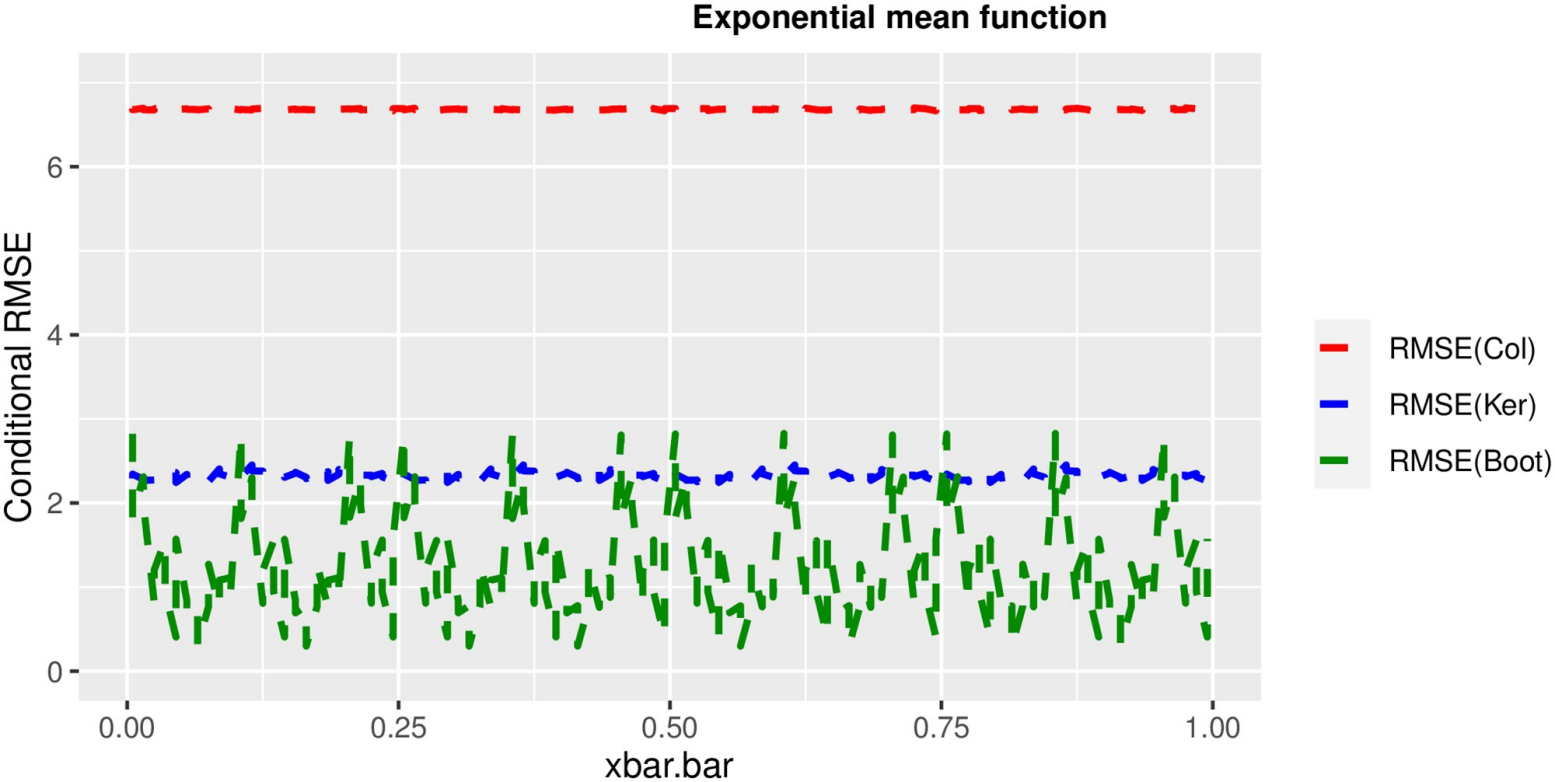
Comparison based on RMSE of the 3 estimators for exponential mean function.

In order to prove the performance of the variance estimates depend on $\bar{x}$, we arranged the 1000 bootstrap samples from each population to increase values in $\bar{x}$. We then grouped the samples in 50 sets of 20 so that the first set contains 20 wherein $\bar{x}$ are smallest, the next set contains the samples with the next 20 smallest in $\bar{x}$, and so on. For each of these so 50 sets, we calculated the average value of $\bar{x}$, the conditional root mean squared error (*CRMSE*), and the variance estimates’ averages for all the variance estimators. Thereafter, the values of *CRMSE* and conditional biases against the average values of $\bar{x}$ was plotted. Figs 1–14 show that the new estimator has a small *RMSE* and biases respectively in almost every scenario considered.

Different strata for all mean functions in deriving the biases were considered. It is clear that the new estimator is better in terms of having small bias under the same conditions than the estimators favoured in the current practice. By comparing the ${\hat{V}}_{ker}$ and ${\hat{V}}_{col}$ estimators, it was found that except for the quadratic, jump and exponential, when smoothing over the discontinuity is the incorrect strategy, the bias of ${\hat{V}}_{ker}$ is substantially smaller than the bias of ${\hat{V}}_{col}$ for any other response variable. By splitting the sample at the discontinuity, calculating two variance estimators, and then combining them, this might be readily solved in reality for both collapsed and kernel variance estimators. In terms of their *RMSE* of the ${\hat{V}}_{ker}$ is small compared to the *RMSE* of ${\hat{V}}_{col}$ except for the jump.

### 5.3 Data application

**Fig 15:**
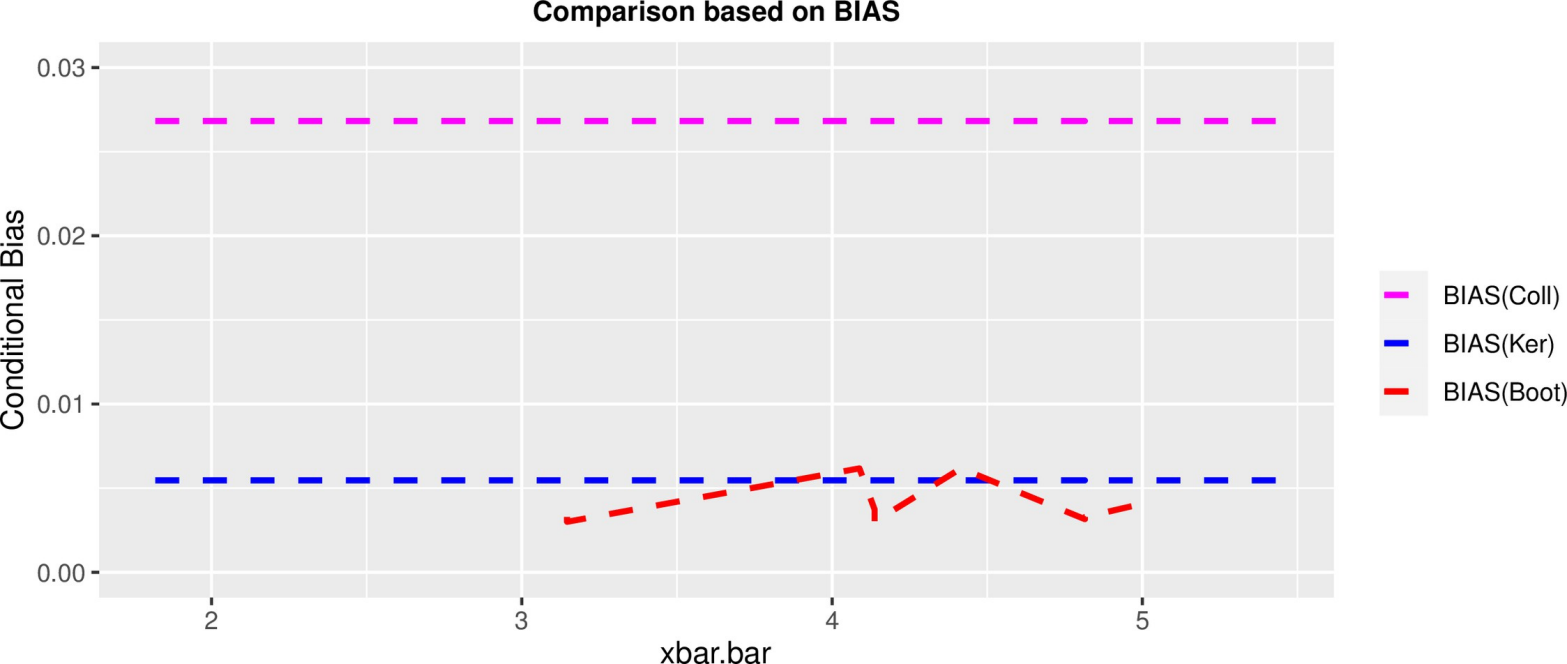
Comparison of the ${\hat{V}}_{boot}$ estimatorag ainst the ${\hat{V}}_{ker}$ and ${\hat{V}}_{cool}$ estimators.

**Fig 16:**
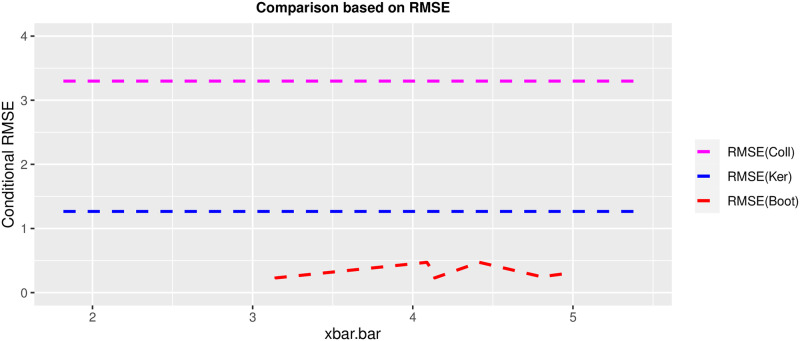
Comparison of the ${\hat{V}}_{boot}$ estimator against the ${\hat{V}}_{ker}$ and ${\hat{V}}_{col}$ estimators.

The performance of the developed estimator is evaluated by using the 1998 SMHO data available in the protocol package in R precisely available on https://rdrr.io/cran/PracTools/man/smho98.html. The Substance Abuse and Mental Health Services Administration in the United States performed the 1998 SMHO. It gathered information on mental health care organizations and general hospitals that provide mental health care services intending to develop national and state-level estimates for total expenditure, full-time equivalent staff, bed count, and total caseload by organization type. *N* = 875 is the original data set size, with 12 variables divided into 16 strata. Then, after eliminating outpatient facilities, only organizations with a number of beds greater than zero remained. After that, the strata pairs {12, 13}, {10, 11}, {6, 8}, and {4, 5} have collapsed due to the small size of the leftover PSUs following the exclusion phase. As a result, we build eight new strata to estimate variance. The number of beds (total inpatient beds) and EXPTOTAL (total expenditures) were considered as the variables of interest, and strata were ordered by *x*_*i*_ = *log* of total beds in stratum *i*. After collapsing the aforementioned strata, a simple random sample without replacement has been used to select a sample size of two PSUs per stratum (*n*_*i*_ = 2) and estimate the variances using the three variance estimators methods specified in the manuscript. Following that, we examine the coefficient of variation (*CV*), root mean squared error (*RMSE*) and the findings for each estimator are shown in the Table 5 as well as in Figs 15 and 16.

**Table 5:** SMOH RMSE, bias and coefficient of variation results.

*H*	*h*	*σ*	Evaluation	${\hat{V}}_{col}$	${\hat{V}}_{ker}$	${\hat{V}}_{boot}$
8	0.143	0.25	*CV*	0.220	0.201	0.004
			*Bias*	0.028	0.0021	0.0011
			*RMSE*	2.405	1.265	0.064
8	0.0625	0.5	*CV*	0.289	0.048	0.006
			*Bias*	0.026	0.011	0.0003
			*RMSE*	1.997	1.947	1.321

The bootstrap-based variance estimator has the lowest *CV* and *RMSE* values as well as the small bias among the rest.

## 6 Concluding remarks

A bootstrap-based variance estimator has been developed as an alternative to the collapsed variance and the non parametric kernel-based variance estimators. These approaches are currently applied in fine stratification and are frequently used in survey statistics research. Fine stratification survey has proved useful in many applications as its point estimator is unbiased and efficient. A common practice to estimate the variance in this context are collapsing the adjacent strata to create pseudo-strata and then estimating the variance, and a non-parametric kernel-based variance estimator but both the attained estimator of variance are not design-unbiased, and the bias increases as the population means of the pseudo-strata become more variant and these estimators may suffer from a large root mean squared error *RMSEs*. A number of alternative variance estimators have been proposed in the literature, but they often rely on some strong auxiliary variables well-correlated with the response variable, or they have a complex form, which make them inapplicable in the real life. This paper proposes a viable solution for this long-standing problem based on a bootstrap-based variance estimator technique that replaces the pseudo strata and the kernel weight by the bootstrap bias corrector. Its properties have been determined, and the simulation study and the real data application show that the new estimator performs well in each case considered. It has a small root mean squared error compared to the current estimators under the same conditions. The proposed approach provides the variance estimates that appropriately account for the complexities of the sampling design and the specific characteristics of interest within the population. It leads to more accurate and precise statistical inference for complex survey data compared to existing approaches.

## Supporting information

S1 File (PDF)

S1 Abbreviation (PDF)
