# Supplementary material for: On bootstrap based variance estimation under fine stratification
Source: PLoS One. 2024 Jun 13;19(6):e0292256. doi: 10.1371/journal.pone.0292256 (PMC11175490; doi:10.1371/journal.pone.0292256)
Supplement: S1 File — (PDF) [file pone.0292256.s001.pdf]

Appendix

Appendix A Prove of Design expectation of the proposed Estimator

$$\begin{aligned} E\big[\hat{V}_{boot}\big] &= E\Bigg[\frac{1}{c_b}\sum_{i=1}^{H}\left(\hat{t}_{bi}-\sum_{j=1}^{H}\hat{a}_{bj}\hat{t}_{bj}\right)^2\Bigg] \\ &= \frac{1}{c_b}\sum_{i=1}^{H}E\left(\hat{t}_{bi}-\sum_{j=1}^{H}\hat{a}_{bj}\hat{t}_{j}\right)^2 \end{aligned} \tag{23}$$

We know that

$$Var(Y) = E(Y^2) - (E(Y))^2$$

This is implies that $E(Y^2) = Var(Y) + (E(Y))^2$
Let $Y = \hat{t}_{bi} - \sum_{j=1}^{H}\hat{a}_{bj}\hat{t}_{bj}$ then,

$$E\big[\hat{V}_{boot}\big] = \frac{1}{c_b}\sum_{i=1}^{H}\Bigg[\left(Var\big(\hat{t}_{bi}-\sum_{j=1}^{H}\hat{a}_{bj}\hat{t}_{bj}\big)\right)+\left(E\big(\hat{t}_{bi}-\sum_{j=1}^{H}\hat{a}_{bj}\hat{t}_{bj}\big)\right)^2\Bigg] \tag{24}$$

But

$$\left(E\Bigg[\hat{t}_{bi}-\sum_{j=1}^{H}\hat{a}_{bj}\hat{t}_{j}\Bigg]\right)^2 = \left(E\big(\hat{t}_{bi}\big)-\sum_{j=1}^{H}E\big(\hat{a}_{bj}\hat{t}_{bj}\big)\right)^2$$

Since $\hat{t}_{bi}$ and $\hat{t}_{bj}$ are approximately unbiased estimators of t_{bi} and t_{bj} respectively, then

$$\begin{aligned} \left(E\Bigg[\hat{t}_{bi}-\sum_{j=1}^{H}\hat{a}_{bj}\hat{t}_{bj}\Bigg]\right)^2 &= \left(E\big(\hat{t}_{bi}\big)-\sum_{j=1}^{H}E\big(\hat{a}_{bj}\hat{t}_{bj}\big)\right)^2 \\ &= \left(t_{bi}-\sum_{j=1}^{H}a_{bj}t_{bj}\right)^2 \end{aligned} \tag{25}$$

Now consider

$$\left(Var\big(\hat{t}_{bi}-\sum_{j=1}^{H}\hat{a}_{bj}\hat{t}_{bj}\big)\right) = Var\big(\hat{t}_{bi}\big)+Var\left(\sum_{j=1}^{H}\hat{a}_{bj}\hat{t}_{bj}\right)-2Cov\left(\hat{t}_{bi},\sum_{j=1}^{H}\hat{a}_{bj}\hat{t}_{bj}\right)$$

As

$$\mathrm{Var}(X-Y)=\mathrm{Var}(X)-2\,\mathrm{Cov}(X,Y)+\mathrm{Var}(Y)$$

Again,

$$\begin{aligned}\left(Var\big(\hat{t}_{bi}-\sum_{j=1}^{H}\hat{a}_{bj}\hat{t}_{bj}\big)\right) &= Var\big(\hat{t}_{bi}\big)+Cov\bigg(\sum_{j=1}^{H}\hat{a}_{bj}\hat{t}_{bj};\sum_{j=1}^{H}\hat{a}_{bj}\hat{t}_{bj}\bigg)\\ &\quad -2Cov\bigg(\sum_{j=1}^{H}\hat{a}_{bj}\hat{t}_{bj},\sum_{j=1}^{H}\hat{a}_{bj}\hat{t}_{bj}\bigg)\end{aligned}$$

because

$$\mathrm{Cov}(X,X)=\mathrm{Var}(X)$$

$$\left(Var\big(\hat{t}_{bi}-\sum_{j=1}^{H}\hat{a}_{bj}\hat{t}_{bj}\big)\right)=Var\big(\hat{t}_{bi}\big)+\sum_{j=1}^{H}\sum_{j=1}^{H}Var(\hat{a}_{bj}\hat{t}_{bj})-2\sum_{j=1}^{H}Cov(\hat{a}_{bj}\hat{t}_{bj},\hat{t}_{bi})$$

The population in which fine stratification is employed have temporal, spatial or other structure that can be exploited in the pairing. If the strata are effectively paired, the bias in collapsed strata is small, in the sense that $t_{bi}\approx t_{bj}$ [3, 6, 9, 15]. The pairing must be done independently of any information obtained from the sample to maintain the statistical properties [6, 9]. Our interest is in estimating the variance of the total, therefore $\hat{a}_{bj}$ is considered as non random normalizing constant depending on resampling but not on survey variable.

For simplicity sake, from (3) , let us assume that
$Var(\hat{t}_{bi})=Var(\hat{t}_i)=V_i$

$$\begin{aligned}\left(Var\big(\hat{t}_{bi}-\sum_{j=1}^{H}\hat{a}_{bj}(\hat{t}_{bj})\big)\right) &= Var\big(\hat{t}_{bi}\big)+\sum_{j=1}^{H}\hat{a}_{bj}^2Var(\hat{t}_{bi})-2\sum_{j=1}^{H}\hat{a}_{bj}Cov(\hat{t}_{bi},\hat{t}_{bi})\\ &= Var(\hat{t}_i)\bigg(1+\sum_{j=1}^{H}\hat{a}_{bj}^2-2\sum_{j=1}^{H}\hat{a}_{bj}\bigg)\end{aligned}$$

Thus,

$$\left(Var\big(\hat{t}_{bi}-\sum_{j=1}^{H}\hat{a}_{bj}\hat{t}_{bj}\big)\right)=V_i\Big[1+\sum_{j=1}^{H}\hat{a}_{bj}^2-2\sum_{j=1}^{H}\hat{a}_{bj}\Big] \tag{26}$$

Now , take (25) and (26) in (24) yields

$$E\big[\hat{V}_{boot}\big]=\frac{1}{c_b}\sum_{i=1}^{H}\left[V_i\Big[1+\sum_{j=1}^{H}\hat{a}_{bj}^2-2\sum_{j=1}^{H}\hat{a}_{bj}\Big]+\bigg(t_{bi}-\sum_{j=1}^{H}a_{bj}t_{bj}\bigg)^2\right] \tag{27}$$

Appendix B The design Variance of the developed estimator

The design variance of $\hat{V}_{boot}$ is given by:

$$Var(\hat{V}_{boot})=E\left[\hat{V}_{boot}^2\right]-\Big(E[\hat{V}_{boot}]\Big)^2$$

Let, $\hat{V}_{boot} = Y$, we have $Var(Y) = E\left[Y^2\right] - (E[Y])^2$ with $E\left[Y^2\right] = Var(Y) + (E[Y])^2$ Now, let us consider term by term:

$$(E[Y])^2 = \left(E\left[\frac{1}{c_b}\sum_{i=1}^{H}\left(\hat{t}_{bi} - \sum_{j=1}^{H}\hat{a}_{bj}\hat{t}_{bj}\right)^2\right]\right)^2 = \frac{1}{c_b^2}\left(\sum_{i=1}^{H} E\left[\hat{t}_{bi} - \sum_{j=1}^{H}\hat{a}_{bj}\hat{t}_{bj}\right]^2\right)^2 \tag{28}$$

Use (24) and (25) in (28) we have

$$(E[Y])^2 = \frac{1}{c_b^2}\left(\sum_{i=1}^{H}\left(V_i\left[1 + \sum_{j=1}^{H}\hat{a}_{bj}^2 - 2\hat{a}_{bj}\right] + \left(t_{bi} - \sum_{j=1}^{H} a_{bj}t_{bj}\right)^2\right)\right)^2 \tag{29}$$

For the next term, we have:

$$\begin{aligned}
Var(Y) &= Var\left(\frac{1}{c_b}\sum_{i=1}^{H}\left(\hat{t}_{bi} - \sum_{j=1}^{H}\hat{a}_{bj}\hat{t}_{bj}\right)^2\right) \\
&= \frac{1}{c_b^2}\sum_{i=1}^{H} Var\left(\hat{t}_{bi} - \sum_{j=1}^{H}\hat{a}_{bj}\hat{t}_{bj}\right)^2 \\
&= \frac{1}{c_b^2}\sum_{i=1}^{H} Var\left(\hat{t}_{bi}^2 - 2\hat{t}_{bi}\sum_{j=1}^{H}\hat{a}_{bj}\hat{t}_{bj} + \left(\sum_{j=1}^{H}\hat{a}_{bj}\hat{t}_{bj}\right)^2\right) \\
&= \frac{1}{c_b^2}\sum_{i=1}^{H}\left(Var(\hat{t}_{bi}^2) - 4\sum_{j=1}^{H}\hat{a}_{bj}^2 Var(\hat{t}_{bi}\hat{t}_{bj}) + \sum_{j=1}^{H}\hat{a}_{bj}^4 Var(\hat{t}_{bj}^2)\right) \\
&= \frac{1}{c_b^2}\sum_{i=1}^{H} V_i^2\left[1 - 4\sum_{j=1}^{H}\hat{a}_{bj}^2 + \sum_{j=1}^{H}\hat{a}_{bj}^4\right]
\end{aligned} \tag{30}$$

Hence the Variance of $\hat{V}_{boot}$ is given by:

$$Var(\hat{V}_{boot}) = \frac{1}{c_b^2}\sum_{i=1}^{H} V_i^2\left[1 - 4\sum_{j=1}^{H}\hat{a}_{bj}^2 + \sum_{j=1}^{H}\hat{a}_{bj}^4\right] \tag{31}$$
