# Supplementary material for: On bootstrap based variance estimation under fine stratification
Source: PLoS One. 2024 Jun 13;19(6):e0292256. doi: 10.1371/journal.pone.0292256 (PMC11175490; doi:10.1371/journal.pone.0292256)
Supplement: S1 Abbreviation — (PDF) [file pone.0292256.s002.pdf]

Abbreviation

$\hat{a}_{bj}$	bootstrap bias corrector at j^{th} stratum
$c_j(i)$	binary function
$d_j(i)$	Kernel weight
CDF	Binary measurement generated from the linear population via $y_k = I_{\{y_{1k} \leq 1.5\}}$
$CHMS$	Canadian Health Measures Survey
$CRMSE$	Conditional Root Mean Squared Error
E	Expectation
h	bandwidth
H	number of strata
I_k	Indicator function
k	sampling unit
MSE	Mean squared Error
N	Population size
PSU	Primary Sample Units
$RMSE$	Root Mean Squared Error
t	total finite population
t_i	population total at stratum i
t_{bj}	bootstrap population total
$\hat{t}$	estimated population total
$\hat{t}_{bj}$	estimated bootstrap population total
U	Finite Population
U_i	population in stratum i
$\hat{V}_{boot}$	Bootstrap based variance estimator
$\hat{V}_{col}$	collapsed variance estimator
V_i	Variance in stratum i
$\hat{V}_i$	estimated Variance in stratum i
$\hat{V}_{ker}$	Non parametric Kernel based variance estimator
y_k	population finite value
π_k	first inclusion probability
